# Health system responsiveness and associated factors among outpatients in primary health care facilities in Ethiopia

**DOI:** 10.1186/s12913-022-07651-w

**Published:** 2022-02-24

**Authors:** Wubshet Debebe Negash, Chalie Tadie Tsehay, Lake Yazachew, Desale Bihonegn Asmamaw, Dawit Zenamarkos Desta, Asmamaw Atnafu

**Affiliations:** 1 Department of Health Systems and Policy, Institute of Public Health, University of Gondar, Gondar, Ethiopia; 2 Department of Reproductive Health, Institute of Public Health, University of Gondar, Gondar, Ethiopia

**Keywords:** Health system responsiveness, Outpatients, Ethiopia

## Abstract

**Background:**

Health system responsiveness is defined as the outcome of designing health facility relationships so that they are familiar and responsive to patients’ universally legitimate expectations. Even though different strategies have been implemented to measure responsiveness, only limited evidence exists in Sub-Saharan Africa. In Ethiopia, information about health system responsiveness among outpatients is limited. Assessing responsiveness could help facilities in improving service delivery based on patient expectations.

**Objective:**

The study aimed to assess health system responsiveness and associated factors among outpatients in primary health care facilities, Asagirt District, Ethiopia, 2021.

**Methods:**

Facility-based cross-sectional quantitative study was implemented between March 30 and April 30/2021. A systematic random sampling technique was employed to select 423 participants, and interviewer-administered data were collected using structured and pretested questionnaires. Both bivariable and multivariable logistic regressions were employed to identify factors associated with health system responsiveness. Adjusted Odds Ratio with their corresponding 95% CI was used to declare factors associated with health system responsiveness. A *p*-value less than 0.05 was used to declare significant statistical variables.

**Results:**

The overall health system responsiveness performance was 66.2% (95% CI: 61.4—70.7). Confidentiality and dignity were the highest responsive domains. Health system responsiveness was higher among satisfied patients (AOR: 9.9, 95% CI: 5.11–19.46), utilized private clinics (AOR: 8.8, 95% CI: 4.32–18.25), and no transport payment (AOR: 1.7, 95% CI: 1.03–2.92) in the study setting.

**Conclusion:**

Overall, health system responsiveness performance was higher than a case-specific study in Ethiopia. To improve the health systems responsiveness and potentially fulfil patients’ legitimate expectations, we need to facilitate informed treatment choice, provide reasonable care within a reasonable time frame, and give patients the option of consulting a specialist. Aside from that, enhancing patient satisfaction, using input from service users, Collaboration, and exchanging experiences between public and private facilities will be important interventions to improve HSR performance.

## Background

All health systems are expected to achieve the goals of good health, responsiveness to the expectations of the population, and fairness of financial contribution [1]. From these goals, health system responsiveness (HSR) is defined by the World Health Organization (WHO) as “how well the health system meets the legitimate expectations of the population for the non-health enhancing aspects of the health system” [2]. Health systems can be evaluated as a whole in any type of interaction by summarizing into responsiveness [3, 4]. The concept entails the experience of people’s fundamental interaction and different factors shaping their interaction with the health system. This intern can be helpful to anticipate and adapt patients’ existing and future health needs for a better health outcome [2, 5].

Responsiveness has been operationalized into eight domains as respect for persons’ dignity; Autonomy to participate in health-related decisions; confidentiality; prompt attention; adequate quality of care; communication; access to social support networks; and Choice of health care providers [5, 6].

Despite the burden of diseases and conflicts in low and middle-income countries [7], providing appropriate and efficient health care delivery and updated health systems with giving attention to intrinsic values and safeguarding patients’ rights are needed [6, 8, 9]. Moreover, the fulfilment of patient expectations is more important than other factors for a better health outcome [10]. Correspondingly, if health system responsiveness has improved, other associated health outcomes have also improved [4].

A patient-centred and acceptable quality across the continuum of care is essential through considering social norms, relationships, values, and trust within societies [11]. Notably, those low and middle-income countries are needed to give attention to equity health access at local and global aspects [12–14]. Equity with good interaction targeting all sections of the society in a health facility is very significant to improve health care utilization [15–17].

The measurement of health system responsiveness helps to evaluate the level of health facilities' performance [1]. Despite challenges for measuring responsiveness, additional refinement of strategy and consistent monitoring are needed to achieve patients' rational expectations [2, 18, 19]. For a better and comprehensive understanding of non-health enhancing aspects of health systems, measuring health care responsiveness is necessary [1, 19]. Additionally, assessing health system responsiveness is needed to improve patients' experience and satisfaction in the sphere of non-medical aspects [15, 20–22]. The reason is that fulfilling patients' expectations are more important than other factors for a better health outcome [10].

A diverse set of factors influence the health care systems responsiveness during the current epidemic crisis, including community factors, socio-economic factors, and environmental factors [23]. Studies revealed that mental illness and medical treatment undermine the dignity and autonomy of the patients [4, 6], in which responsiveness is increasingly essential for such patients [24]. Client satisfaction with the health system and quality of care have fundamental importance in managing interpersonal interaction [4]. Existing literatures in Ethiopia revealed that patients' good satisfaction with the provision of health care services [25, 26] and perceived quality of care about the services they received and the patient values and interests in the services [25] were associated with health system responsiveness positively. The above factors create gaps in the responsiveness performance to meet the expectation of the clients regarding how they should be treated and the convenience of the environment in which they are treated [25].

Generally, improving responsiveness needs performance evaluation and higher spending level from a policy perspective in low-income settings [16, 27]. To encourage this, Ethiopia has a vision towards UHC by strengthening primary health care by 2035. The country is now implementing the second health sector transformation plan (HSTP-II) for the next five years from July 2020 [28]. In this plan, responsiveness is one of the key priority objectives to improve patients’ preferences, needs, and values during health service provisions relative to patients’ non-medical needs [17, 28]. The measurement and evidence of responsiveness performance can be used by governments to re-evaluate the health reforms [8, 29] and can help more effective service utilization [5]. To promote this, the Ethiopian government recommends the health sectors and researchers explore information on responsiveness and efforts to meet attributes in the coming strategic period [28].

Although WHO has a strong commitment on the implementation of the strategy for evaluating responsiveness, the measurement is still challenging [2]. Due to little evidence on the health system responsiveness in the primary health care settings [30], there is a need to interview patients to know their experiences with the health system responsiveness [29]. However, little is known in African countries [16, 31, 32]. Particularly, in Ethiopia, there is no systematically organized study addressing health system responsiveness in a domain-based manner. Therefore, this study was aimed to fill this research gap by assessing health system responsiveness and associated factors among outpatients from primary health care facilities.

## Methods

### Study design, setting and period

A facility-based cross-sectional quantitative study design was conducted to assess health system responsiveness among outpatients from March 30 to April 30 /2021. The study was conducted in Asagirt District, North Shewa Zone, Ethiopia. The District is 125.5 Kilometers (Km) far from Addis Ababa, the capital city of Ethiopia. It has 15 kebeles (the lowest administrative units). The 2020 projected population of the District was 57,320. Of whom 30,240 were males. The District has 20 functional health facilities: 3 public health centres, 2 primary private clinics, and 15 health posts (community-level health facilities providing basic preventive and medical care). In 2021 a total of 52 health professionals and 23 health extension workers were served the District. According to the District health managers’ report, there was an average of 1700 patients visiting health centres and private clinics within a month.

### Sample size, sampling and participant selection

The sample size for outpatients to participate in the study was determined by using single population proportion formula [33]. With an assumption of a 50.0% proportion (there is no local data available on the subject for outpatient and to get maximum sample size), 95% of confidence level $({\mathrm{Z}}_{\mathrm{\alpha }/2}=\hspace{0.17em}1.96)$, margin of error = 5%. After adding a 10% non-response rate the total sample size was estimated to be 423 clients. Computed as $\mathrm{n}=\frac{{({\mathrm{Z}}_{\frac{\mathrm{\alpha }}{2}})}^{2}\mathrm{P}(1-\mathrm{P})}{{\mathrm{d}}^{2}}$

$$\mathrm{n}=\frac{{(1.96)}^{2}0.5(1-0.5)}{{(0.05)}^{2}}=384.16$$

$$\mathrm{n}=384.16+38.416=423$$

**Fig. 1:**
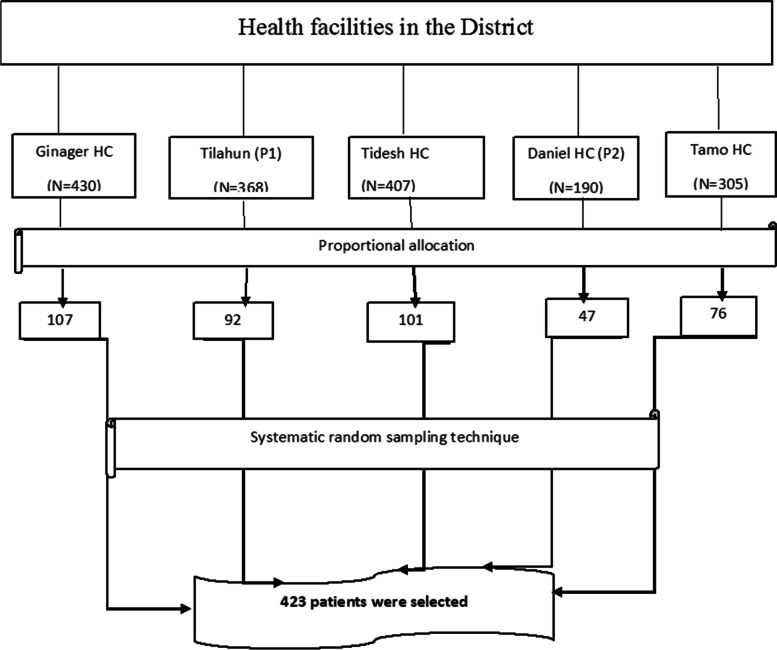
Schematic presentation of the sampling procedure to select study participants

The distribution of samples throughout the health facilities was determined by the probability proportional to their size. A systematic random sampling technique from all five primary health care facilities was employed to select the calculated sample size. Then at every k^th^ interval (K = N/n) where *N* = total clients who have received health care services within the study period *n* = required sample size, thus K = 1700/423 = 4. Then, the first patient was randomly identified from 4 by lottery method. Then every 4^th^ patient was taken into the study until the required number of study participants for each facility in the outpatient department was reached (Fig. 1).

All outpatients who received health care services in primary health care facilities residing in the District constitute the source population of this study. Patients who received health care services as an outpatient in the selected primary health care facilities were included in the study. Patients aged below 18 years and all outpatients who visited the health posts were excluded from this study.

### Data collection tools and variables

Closed-ended interview questionnaires adapted from WHO health system responsiveness and from different related literatures, were used for data collection. The questionnaire mainly includes socio-demographic variables (age, sex, educational status, marital status, occupation, household’s monthly income), health service accessibility-related factors (time to reach health facility, type of health facility, out of pocket payment for transport to reach the health facility), WHO responsiveness assessment questionnaires, and finally individual related factors like perceived quality of health care, perceived satisfaction. The questionnaire was prepared in English first, then translated to Amharic (local language), and then retranslated back to the English language to check its consistency. The data collectors collected the data systematically after the patients received the services on their way to the home (exit interview).

Health system responsiveness of outpatient health care service was the dependent variable. It was assessed by 28 items customized from WHO multi-country studies and the report of Ethiopia’s health sector transformation plan (HSTP II) [4, 24, 27, 34]. The 28 items was divided among seven domains as communication (4), Confidentiality (3), Quality of basic amenities (5), Dignity (4), Choice (3), Prompt attention (5), Autonomy (4). The eighth domain (access to social support network) was not assessed since it is used for evaluating inpatients (hospitalization) only [29, 35] (Table 1).

**Table 1:** Responsiveness sample questions under each domain and item properties in the interview

Domains	Sample questions	Answer categories
Prompt Attention	How would you rate the length of time spent at health care units waiting for consultation/treatment reasonable?	1. Never 2. Only sometimes 3. Usually 4. Always
Dignity/ respect	How often did doctors, nurses, or other health care providers treat you with respect?	
Communication	How would you rate the health care provider’s explanation of things in a way you could understand?	
Autonomy	How often did health care providers involve you in deciding about the care, treatment, or test?	
Confidentiality of information	How often talked with your health care provider done privately so other people who you did not want to hear could not overhear what was said?	
Choice	How often health care providers did you have a choice between health care providers in the health care unit?	
Quality of surroundings/ amenities	How would you rate the basic quality of the waiting room, for example, space, sitting, and fresh air?	1. Very poor 2. poor 3. good 4. very good

All the 28 items were computed and then dichotomized as “acceptable” and “unacceptable” by the demarcation threshold formula as:$\frac{{\varvec{T}}{\varvec{o}}{\varvec{t}}{\varvec{a}}{\varvec{l}}\boldsymbol{ }{\varvec{h}}{\varvec{i}}{\varvec{g}}{\varvec{h}}{\varvec{e}}{\varvec{s}}{\varvec{t}}\boldsymbol{ }{\varvec{s}}{\varvec{c}}{\varvec{o}}{\varvec{r}}{\varvec{e}}-{\varvec{T}}{\varvec{o}}{\varvec{t}}{\varvec{a}}{\varvec{l}}\boldsymbol{ }{\varvec{l}}{\varvec{o}}{\varvec{w}}{\varvec{e}}{\varvec{s}}{\varvec{t}}\boldsymbol{ }{\varvec{s}}{\varvec{c}}{\varvec{o}}{\varvec{r}}{\varvec{e}}}{2}) +\mathrm{total lowest score}$ [26, 36, 37]. Accordingly, those who scored 73 and above HSR were considered as “Acceptable” and below considered “Unacceptable”.

Likewise, all the seven domains were added separately and grouped as good and poor by the above formula [26, 36, 37]. Above the cut-off point to determine “Good” performance, while including cut-off point and below scores, was considered “Poor” for each domain independently. Cronbach’s alpha reliability test checked the reliability of the tools. Accordingly, average Cronbach’s alpha for all domains was 0.92, all showed high internal consistency above the required cut-off 0.70.

Patient satisfaction was measured using 5 questions on a five-point Likert scale with five response categories (1 ‘very dissatisfied’ to 5’ very satisfied’). Then, it was grouped using the demarcation threshold formula [36, 37]. Accordingly, those who scored 15 and above were considered as “Satisfied” whereas below 15 was considered “Dissatisfied”. Similarly, Perceived quality of care score was assessed by 12 questions, Then it was dichotomized into “high” for those who scored above 37 and “low” for those who scored 37 and less [26]. Patient health quality (PHQ-9) was assessed by 9 depression questions ranging from 1 ‘always’ to 4 ‘not at all’ after which it was dichotomized as “poor” and “good” with a cut-off point of 23 [38].

Internal consistencies of Cronbach’s alpha values for patient health quality, satisfaction, and quality of health care were 0.87, 0.89, and 0.96 respectively and were all above the required cut-off of 0.70.

**Fig. 2:**
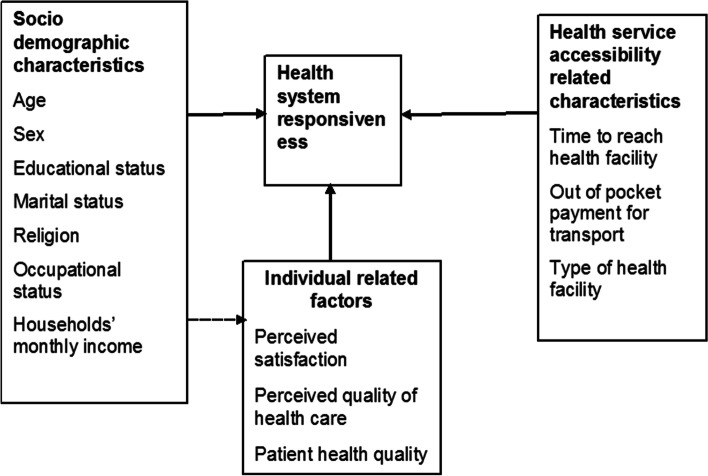
Conceptual framework developed from different literatures

The conceptual framework summarized different factors to assist in exploring factors associated with the health system responsiveness from different works of literature, including socio-demographic characteristics, health service accessibility related factors, patient satisfaction, and quality of healthcare-related factors. The framework was adapted from different literatures [25, 39–43] (Fig. 2).

### Data quality assurance

Data was collected by five B.Sc. Health Officers and two supervisors from the same field working out of study areas after they were trained in how to collect the data. Furthermore, the facility workers were not permitted to see or hear the patients’ responses. Before starting the actual data collection, the data collectors performed field practice and pretested the questionnaires on 21 (5%) individuals. The data collection tool was modified based on insights and experiences gained from the pretest (As an example, the Likert scale for the response categories for the dependent variable was modified from 5 to 4 options). In the event of any problems, the investigator discussed them with the supervisor daily and made corrections.

### Data processing and analysis

The data were checked for completeness and entered into the Epi-data version 4.6 Software Package. Then it was exported to Stata version 14 statistical software packages for cleaning, coding, and analysis. A two-stage data analysis (descriptive and inferential) was conducted. The descriptive statistics were described using frequency, percentage, mean and standard deviation and presented by a figure, table, and text. All continuous independent variables were categorized. Normality tests such as kurtosis and skewness were employed to identify which summary measure is appropriate to use. Multicollinearity among independent variables was checked using variance inflation factor (VIF) and was found no multicollinearity (mean value = 1.13). Both bi-variable and multivariable logistic regressions were employed. All explanatory variables in bivariable analysis with a *p*-value of 0.25 and less were considered candidate variables for multivariable analysis to control confounding factors. The final model used Adjusted Odds Ratio (AOR) with their corresponding 95% confidence intervals (CI) to declare factors associated with health system responsiveness. A *p*-value less than 0.05 was used to report statistical significance in this study.

## Results

### Socio-demographic characteristics of the study participants

In this study, a total of 417 outpatients were interviewed. The median age of the study participants was 19 (IQR: 25–49) years. About 40.8% of participants aged 18–29 years, more than two-thirds (69.6%) were rural dwellers. The majority (92.8%) were Orthodox Christian followers in religion (Table 2).

**Table 2:** Respondents’ socio-demographic characteristics (*n* = 417)

Variables	Frequencies (*n*)	Percentage (%)
Sex		
Male	226	54.2
Female	191	45.8
Age (years)		
18–29	170	40.8
30–39	89	21.3
40–49	54	13.0
50 and above	104	24.9
Residence		
Rural	288	69.6
Urban	129	30.4
Religion		
Orthodox	387	92.8
Muslim	30	7.2
Occupational status		
Farmer	254	60.9
Government employee	53	12.7
Merchant	45	10.8
Others^a^	65	15.6
Current marital status		
Married	255	61.1
Not married^b^	162	38.9
Educational status		
Unable to read and write	70	16.8
Able to read and write	105	25.2
Primary (grade 1–8)	135	32.4
High school and above	107	25.6
Household monthly income(ETB)^c^		
> 650	138	33.1
< = 650	268	64.3
Unknown	11	2.6

^a^Student, private employee, daily laborer, ^b^Single, divorced, windowed, ^c^Ethiopian Birr (currency)

### Health service accessibility-related characteristics

More than two-thirds (67.6%) of the participants utilized health services from public health facilities. More than half (56.1%) travelled one hour or less to reach the health care facility (Table 3).

**Table 3:** Health facility accessibility-related characteristics of the study participants (*n* = 417)

Variables	Frequencies (*n*)	Percentage (%)
Time to reach the health facility on foot		
= < 1 h	234	56.1
> 1 h	183	43.9
Type of health facility		
Public	282	67.6
Private	135	32.4
Visited traditional healer		
Yes	185	44.4
No	232	55.6
OOP payment for transport^a^		
Yes	205	49.2
No	212	50.8

^a^Out of pocket

### Patient-related characteristics

Most (81.3%) of the respondents had good satisfaction. Regarding patient health quality (PHQ-9), more than three-fourths (84.6%) had good perceived patient health quality (Table 4).

**Table 4:** Patient related characteristics of the study participants (*n* = 417)

Variables	Frequency (*n*)	Percentage (%)
Perceived satisfaction		
Satisfied	339	81.3
Dissatisfied	78	18.7
Perceived health care		
High	338	81
Low	79	19
PHQ9^a^		
Good	353	84.6
Poor	64	15.4

^a^Patient health quality

### Performance of health system responsiveness

**Fig. 3:**
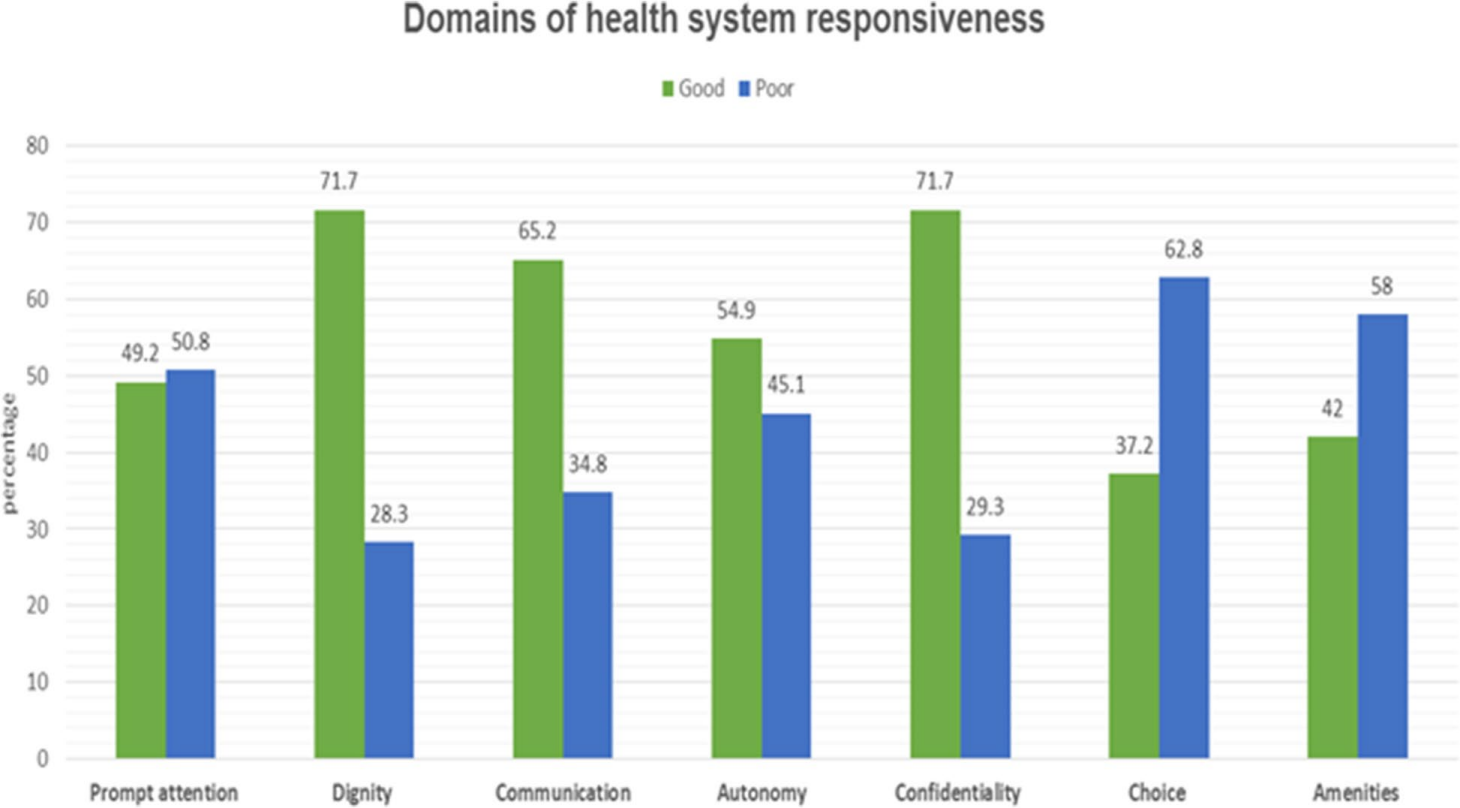
Health system responsiveness of the respondents

The overall performance of health system responsiveness was 66.2% (95% CI: 61.4–70.7). The performance of both confidentiality and dignity was nearly 72%, which is good performance. On the other hand, the Choice domain was the least (37.2%) scored on the good category of performance (Fig. 3).

### Factors associated with health system responsiveness

Binary logistic regression was employed to evaluate the association between different socio-demographic, health facility-related, and patient-related variables with health system responsiveness. Variables with a *p*-value < 0.25 in the bivariable analysis were considered candidates for multivariable analysis. Accordingly, age, occupation, educational status, type of facility, out of pocket payment for transport, perceived satisfaction of care, and perceived quality of health were selected. Model fitness was tested with Hosmer and Lemeshow Goodness of Fit test (*p* = 0.52). In the final multivariable logistic regression analysis: type of health facility, OOP payment for transport, and perceived satisfaction were significantly associated with HSR performance.

Thus, the odds of health system responsiveness among outpatients who utilized private health care facilities were 8.8 (AOR: 8.8, 95% CI: 4.32– 18.25) times higher than those who utilized health care services from public health facilities. Patients who had not paid for transport to reach the health facilities had 1.7 times higher odds of health system responsiveness than their counterparts (AOR: 1.7, 95% CI: 1.03 – 2.92). The likelihood of health system responsiveness among satisfied patients was nearly 10 times higher when compared with those outpatients who had poor satisfaction (AOR: 9.9, 95% CI: 5.11–19.46) (Table 5).

**Table 5:** Multivariable analysis of factors associated with HSR in primary health care facilities, Asagirt District, Ethiopia, 2021(*n* = 417)

Variables	Health system responsiveness		COR (95%CI)	AOR (95%CI)
	Unacceptable	Acceptable		
Age in years				
18–29	52	118	1	1
30–39	39	50	0.56 (0.33–0.96)	0.92 (0.47– 1.80)
40–49	19	35	0.81 (0.43–1.55)	1.00 (0.45–2.0)
50 and above	31	73	1.04 (0.61–1.77)	1.64 (0.82–3.29)
Occupational status				
Farmer	56	101	1	1
House wife	35	62	0.98(0.58–1.66)	0.98(0.51–1.87)
Employed	30	88	1.63(0.96–2.76)	1.03(0.46–2.32)
Merchant	20	25	0.69(0.35–1.36)	0.77(0.33–1.79)
Educational status				
Unable to read and write	25	45	1	1
Able to read and write	36	69	1.06 (0.57–2.00)	0.85 (0.39–1.82)
Primary (Grade 1–8)	58	77	0.74 (0.41–1.34)	0.51 (0.24–1.05)
High school and above	22	85	2.15 (1.09–4.22)	1.21 (0.44–3.31)
Type of health Facility				
Public	129	153	1	1
Private	12	123	8.64 (4.57–16.35)	8.88 (4.32–18.25)***
Out of pocket expense for transport				
Yes	85	120	1	**1**
No	56	156	1.97 (1.31–2.98)	1.74 (1.03–2.92)**
Patient health quality				
Good	113	240	1.65(0.96–2.84)	0.80(0.39–1.62)
Poor	28	36	1	1
Patient satisfaction				
Dissatisfied	61	17	1	**1**
Satisfied	80	259	11.62 (6.42–21.02)	9.98(5.11–19.46)***

^*^ Significant at *P* < 0.05, ** Significant at *P* < 0.01, *** Significant at *P* < 0.001

## Discussion

This study was carried out to determine the level of health system responsiveness and to identify factors associated with the health system responsiveness among outpatients in the primary health care facilities in Asagirt District, North Shewa Zone, Ethiopia. The study highlighted that the health system was responsive for nearly two-thirds (66.2%) of health care users. The finding indicated that a large proportion (33.8%) of patients need a more responsive health system on the contrary.

In this study, the finding of the HSR performance is consistent with a study conducted in Wolaita zone, Ethiopia, in which the performance of health system responsiveness was 68.3% [25]. However, this result was higher than the report of the Federal Ministry of Health (FMOH) in service responsiveness normalized score (52%) [19]. The possible explanation for this difference might be that the result from the FMOH was only an average report or the adjusted values measured on different scales to a common scale.

Similarly, the result was higher than a study conducted in Shewarobit, Ethiopia, in which 55.3% of the health system responsiveness was good performance [26]. The higher responsiveness could probably difference in the study participants; in this study, we investigated HSR among all outpatients from each primary health care facility in the District; however, in Shewarobit, the study was conducted on case-specific responsiveness among HIV positive individuals. Additionally, the observed better responsiveness performance may result from the government’s ongoing efforts to improve service delivery.

On the contrary, the finding was lower than a study conducted in Brazil (80%) [35]. This is possibly due to the differences in health care availability and accessibility where there is better availability and continuity of primary health cares in Brazil [44]. Probably also the difference in socio-demographic characteristics of the study participants. In Brazil, it was conducted among older adults aged 60 and above years, whereas in our study, the participants were aged from 18 years, and nearly 41% were below 30 years.

This study revealed that the health system responsiveness has differed across the domains. The finding is supported by studies conducted in Iran [45], Brazil [35], and Ethiopia [26]. Accordingly, of the seven domains which were measured, confidentiality (privacy) (71.7%) and dignity (respect) (71.7%) had performed better than other domains. This is in line with two studies conducted in Iran [45, 46]. Similarly, in Tanzania, confidentiality (86.7%) and dignity (81.4%) were the highest scores from the domains of responsiveness [15]. The higher score for the two domains might be users’ high expectation of privacy and safeguard of personal information by a health professional [47].

From the findings of this research, the domain of Choice (of health care providers and units) was found to be the lowest good performance (37%) among outpatient health care services. This is in line with a study conducted in Iran [45] that reported the lowest good performance (35.8%) in the choice domain. However slightly better than a study conducted in Brazil, which was 24.4% [35]. The possible explanation for this difference might be explained as the study period such that the study conducted in Brazil was seven years back. From then, many improvements might have taken place. Therefore, an adequate staff proportionate to the population needs is essential while planning health professional recruitment for the District.

Despite the highest responsiveness performance scores on confidentiality (privacy), dignity (respect), communication (interaction with service providers) domains, our results revealed a concern by patients regarding the domains of prompt attention (waiting time) and basic amenities (convenience of facilities). These findings are similar to previous studies conducted on health care responsiveness in South Africa [29], Nigeria [32], and Tanzania [31], in which prompt attention and quality of basic amenities were poorly performed. Therefore, demand and supply investments and increasing the physical structure of the units proportional to the District population are needed.

This study revealed that patients’ satisfaction, type of health facility, and out-of-pocket payment for transport to reach the health facility were identified as factors affecting the performance of health system responsiveness.

From the findings of this study, overall, HSR did not significantly associate with the socio-demographic backgrounds of the study participants. This is in line with two other case-specific studies conducted among HIV-positive individuals in Ethiopia [25, 26]. This might indicate that HSR does not differ by socio-demographic background [25]. The non-association might require further exploration. On the contrary, a study in Nigeria [47] found that gender, educational status, and income were significantly associated with the performance of HSR. Similar to this in Tanzania [31], older age, sex, and being married were associated negatively whereas, high income and educational status were positively associated with responsiveness. Elsewhere studies in Germany [48], Thailand [21], Iran [49], and India [50], age was significantly associated with the health system responsiveness.

Health system responsiveness depends on the financial aspects of health care [51]. WHO suggested that when patients travel a long time to get medical services, they will poorly evaluate the health system responsiveness [52]. Similarly, our findings showed that the odds of HSR among participants with no out of pocket payment for transport to reach the health facility was 1.7 times higher than its counterparts. This could probably be because the rating of HSR might be influenced by the expectations against relative total worth of expense in obtaining needed health care. As financial fairness improved, customers rated health facilities more responsive [25].

The findings of this study have clearly shown that the likelihood of HSR among participants who were utilized private health facilities was nearly nine times higher than participants who used public health facilities. Similar to this, findings from the African countries of Ghana [53], South Africa [29], and Iran [49] revealed that the health system responsiveness of public health facilities was found lower as compared to private health care facilities. The possible reason for the highest responsiveness in private facilities might be that private facilities aim to maximize their profit. To achieve this profit, they are more responsive to attract (satisfy) clients. In addition, ownership of health care is a factor for better performance [43]. Therefore, it might be better if private health care facilities share their experience for public health facilities.

When clients are dissatisfied with health outcomes, responsiveness mean sum scores will become low [3, 42]. In agreement with this idea, this study observed that clients who had good satisfaction with the health care they offered had higher HSR than poorly satisfied individuals. Elsewhere studies in Ghana, Ethiopia [16, 25, 26] also indicated that the more satisfaction, the higher the responsiveness. Additionally, the world health organization also suggested that all health system responsiveness domains were positively and significantly related to satisfaction [54]. Perhaps because patients are satisfied with a non-medical aspect of care, they become better compliant and understand all the interactions of results [37]. Therefore, health facility administrators should continuously look for new ways such as feedback from patients to increase patient satisfaction scores as efficiently and effectively as possible.

### Strengths and limitations

The time gap between service received by participants and data collection was on the same day. Thus it is unlikely to have a significant level of recall bias. We also acknowledge the response bias because of the self-reported data. To reduce the bias, short and interval questionnaires were employed. With regard to the limitations, the data were collected only from the patient perspective or did not include the providers’ perspective. It could be better if the research was performed with a mixed approach. Because of the cross-sectional nature, causal relationships between the associated factors with health system responsiveness cannot be established.

## Conclusion

This study contributes to health system responsiveness research in Ethiopia among outpatients at primary health care facilities. Even though relatively higher health system responsiveness than case-specific study in Ethiopia, the result showed that only confidentiality and dignity domains found the highest score. Overall, HSR was higher in private than public healthcare facilities. Additionally, satisfied clients and those who didn’t pay for transport on their way to the health facility were better responsive than their counterparts. The domain of Autonomy, Waiting time, Basic amenities, Choice were identified as inadequate to meet the legitimate expectation of the clients regarding the non-health aspects of medical care. They need the effort to raise responsiveness of health care service in the District. In addition to this, health facility administrators must enhance patients’ satisfaction by using inputs from service users. Sharing experience, collaborating with private clinics, and giving attention to distant coming patients will be essential interventions to improve HSR.

## Data Availability

The data set is available on a reasonable request from the corresponding author.

## Abbreviations

AOR: Adjusted Odds Ratio
CBHI: Community Based Health Insurance
CI: Confidence Intervals
COR: Crude Odds Ratio
FMOH: Federal Ministry of Health
HIS: Health Insurance Scheme
HSR: Health System Responsiveness
MCSS: Multi-Country Survey Study
NHIS: National Health Insurance Scheme
OR: Odds Ratio
PHQ: Patient Health Quality
SHI: Social Health Insurance
UHC: Universal Health Coverage
VIF: Variance Inflation Factor
WHO: World Health Organization
